# Medical News and Miscellany

**Published:** 1887-08

**Authors:** 


### MEDICAL NEWS AND MISCELLANY.

FOR SALE.—At this office, a Caligraph, No. 1 (capitals and lowercase type, two alphabets), used but little, as good as new, and in perfect order—will be sold for $55; cost price at factory $85. Also, "The World Type-Writer," price $8; or for nickel-plate, in leather case, $10—a good machine, no toy, but does good and fast work, and can be carried in satchel, on cars, or in buggy. The very thing for writing labels. It is strong, and does not get out of order. Speed equal to any machine.

DISCONTINUED.—The *New York Medical Monthly*, Dr. Corning, editor, has suspended publication.

---

THE *New York Medical Record* will have stenographers in every section at the Congress, and offers to furnish medical journals with reports free of charge.

---

NOTICE TO MEMBERS T. S. M. A.—The Transactions for 1887 will be in paper covers. The printer, by miscount, ran off fifty or sixty copies more than were ordered, and the Publishing Committee refuse to pay for them. Under the circumstances, he has had them bound in handsome cloth covers, like those of last year, and to "get his money back" offers to sell them at cost—$1 per vol.

---

ANOTHER ASYLUM QUARREL.

Dr. R. S. Johnson has preferred charges against Dr. Dorsett, superintendent of the Lunatic Asylum, the specifications covering, in general, mismanagement of the institution. Messrs. Von Resenberg & Wheless have been retained by Dr. Johnson to prosecute the charges before the board of managers of the asylum.—*Statesman.*

---

If ever I go to the war,
I'll go in the medical corps,
And then while they're fighting, and biting, and smiting, and shedding bad language and gore,
I'll turn from the strife I abhor,
Both sides of the field I'll explore,
Where the wounded are creeping, and sleeping, and weeping, sweet balm in their hurts I will pour.

—*Bob Burdette.*

---

THE UNIVERSITY OF TEXAS AND THE A. & M. COLLEGE.—There is an unfortunate misunderstanding between the Regents of the University of Texas and the Directors of the A. & M. College. The A. & M. College, though under separate management, is a part of the University—yet received Federal aid independently. Dr. Wooten, President of the Regents, in his address before the State Medical Association (published in our June issue), gave his version of the controversy, and indulged in some strictures on the latter institution. This has called forth a reply from Mr. Garrett, President of the Directors of the A. & M. College, a very mild and conservative document, in which he begs to correct certain statements made by Dr. Wooten with reference to the matter. It seems that the Legislature gave the A. & M. College one-fifth of the University fund for two years, while the Regents felt that they should have the disbursing of the entire fund, the A. & M. being but a branch of the main institution. There is, unfortunately, a prospect of a pretty lively controversy, and it is feared it will come up at the next State Medical Association meeting.

---

The election of Dr. Dudley S. Reynolds to the presidency of the Mississippi Valley Medical Association for the coming year was a graceful recognition of his services to the Association and of his personal and professional standing. In the election it was a high compliment that his strongest support came from his own city.

Dr. Matthews of Louisville, is a man whom to know is to admire. His brave and generous defence of his friend against a recent scurrillous attack brought down the house. A few such men as Mathews in each city would to a large degree stamp out all professional jealousy and strife.—*Weekly Medical Review.*

Dr. Reynolds has been made one of the Vice-Presidents of the Section on Ophthalmology in the International Medical Journal.

---

A Newspaper in Illinois recently brought suit against forty-three men who would not pay their subscriptions, and obtained judgment in each for the full amount of the claim. Of these, twenty-eight men made affidavit that they owned no more property than the law allowed them, thus preventing attachments. Then they, under the decision of the Supreme Court, were arrested for petty larceny, and bound over in the sum of $300 each. All but six gave bonds, while six went to jail. The postal law makes it larceny to take a newspaper and refuse to pay for it.—*Atlanta Medical and Surgical Journal.*

We have perhaps fifty instances where parties have taken this Journal a year, and refused to pay for it, because they "did not subscribe."

---

MEDICAL NEWS.—This journal is intended as a medium through which all news and information of interest to the profession in Texas shall be disseminated; it aims to keep its readers and the profession, generally, posted as to what is going on the medical circles in Texas, and we flatter ourselves that the object is pretty well accomplished. As illustrating the necessity of subscribing for the JOURNAL, and thus keeping posted, we will state that, although in our last number we gave a full account of the present status, and future prospect, of the Texas Medical College at Galveston, we received to-day a letter addressed to *us* as Dean of the Texas Medical College, *Austin*. Of course, it is unnecessary to say this doctor is not a subscriber; he had heard rumors of a college, somewhere, but he was not posted. Subscribe at once; the third volume is just begun.

---

WE WOULD call attention to the unique case reported by Dr. Menger, "Thrash" as it is commonly called has always been considered a harmless ailment, and so far as we know such a thing as a fatal hemorrhage resulting from it is unheard of. We are inclined to the belief that there was a hemorrhagic tendency or diathesis, and that the hemorrhage was really produced by the rubing with what the Doctor says was "Salvia tea." Sage tea is harmless, but it is probable that in this instance there was some irritant substance accidentally mixed with it, perhaps in the gathering of the herb. We would like to hear from our readers on the subject.

---

DR. T. J. TYNER, of Austin, entertained a select party of medical friends at a royal dinner at the Driskill on the eve of his departure for New York—"a really scientific dinner," said the Nestor of Texas surgery, our own Cupples, who did our popular and esteemed host the honor to grace the board on that occasion, coming from San Antonio for the purpose. Dr. Tyner will take a special course at the Post Graduate School, or the Polyclinic, visit all the hospitals and dispensaries, learn all that is new in his special branch, as well as in general medicine and surgery, and run down to Washington during International Congress week, and take an active part in the work in the ophthalmological section. His health was drank in bumpers of choicest wines, and many were the cordial wishes for his safe and speedy return. Few medical men have such a hold on the affections and esteem of their colleagues as Dr. T. J. Tyner. Success to him!

Dr. O. L. Abney, of Goliad, is attending a special course at the N. Y. Post Graduate Medical College, New York.

---

Died—In Dallas, Texas, July 18, ult., Dr. Jno. H. Morton, a prominent physician and old practitioner of that city. He was a member of the Texas State Medical Association.

---

Dr. Wm. Caston, of Corsicana, will read a paper on "Cleft Palate," at the coming meeting of the American Rhinological Association in Washington, D. C. on Saturday, Sept. 3d, prox.

---

Key West—Yellow Fever.—The medical officer in charge of the Marine Hospital Service (Passed Assistant Surgeon John Guiteras) reports a total of 208 cases and 44 deaths up to Aug. 4th.

---

On dit that our talented young friend Dr. Dabney, of Creath & Dabney, Kinneyville, Texas, will lead to the altar one of Louisiana's fairest daughters, on, or about, the 20th inst. "Blessing on thee, my children."

---

Death from Ether.—In Philadelphia, the other day, a Mr. Dill died while ether was being administered to him for some surgical purpose, by Dr. Agnew. "Accidents will happen"—"no blame attaches to the doctors."

---

The Paper by Dr. Wallace (See "*Society Notes*") read before the Terrell Medical Society, is a classical production—notwith standing the Doctor disclaims all attempt at style. It is one of the best contributions we have yet had, and every physician, and especially, every society member can read it with benefit.

---

The Association of American Medical Editors will entertain the editors of foreign medical journals at a $3,000 banquet, on Monday evening, Sept. 5, in Washington City. "The ticket has been fixed at $15 a plate," says a neat little notice we received, just following one informing us that we had been appointed "a member of the committee of arrangements" for the meeting.

---

The New District Medical Society.—In response to the circular sent out to physicians in Travis and adjoining counties—proposing the organization of a new district medical society—it has been agreed to hold a meeting at Austin on the 8th of September. A large number of physicians have responded, and promised to be present, and the proposition appears to meet with very general favor. Do not forget the date. Come and join us. Preparations will be made for a "good time"—socially.

---

"Dr. W. C. Wile and Lady," is the way it will be registered at the Arlington.

Oh, the Wiley Wile! He now Loomis up as a prospective Benedict. His friends knew that he had always Benedict(ed) to an admiration of the ladies, and now he is to be Benedicted "sho nuff." "Who'd ha' thought it?" "Just as we expected." He has called for (Adele), a new deal, and we trust may soon enter into a (New) Haven of happiness. This accounts for the Doctor's wonderful energy and success. Anybody might have suspected he was in love, for love is the most powerful inspiration to help a man on to success in any sphere of action. We acknowledge the courtesy of an invitation, and tender our congratulations.

"Mr. and Mrs. W. C. Loomis—marriage of their daughter, Hattie Adele, to Dr. W. C. Wile—Methodist Church, New Haven, Connecticut, 7 p. m., September 1, '87."

---

ADDITIONAL DELEGATES T. S. M. A. TO INTERNATIONAL MEDICAL CONGRESS.

After the list of delegates as furnished the Secretary by the nominating committee had been published in the Transactions and in this Journal the following list was found on the fly-leaf of the Register, in the hand-writing of the Clerk (Mr. Dixon) who assisted the Treasurer at the late meeting; and the Secretary, has issued certificates to the following members, in addition to those whose names have heretofore been published: Drs. C. F. Paine, J. M. Willis, A. M. Douglass, J. P. Oliver, O. L. Williams, F. H. Tucker, W. B. Brooks, J. C. King, Fred. Terrell, T. D. Wooten, W. Caston, (vice F. E. Daniel.)

The Secretary has no right to make any additional appointments of course, and the President has decided that the only way a certificate as delegate can be had now, is to arrange with some appointee, who is not going, to send in his resignation in favor of applicant, The President will then instruct the Secretary to issue certificate in his stead. This arrangement was made between Dr. Daniel (delegate), and Dr. Caston, who wanted to go.

